# Dynamics and Stability Mechanism of Lactoferrin–EPA During Emulsification Process: Insights from Macroscopic and Molecular Perspectives

**DOI:** 10.3390/foods14010082

**Published:** 2025-01-01

**Authors:** Han Tao, Wei Ding, Meng-Jia Fang, Hao Qian, Wan-Hao Cai, Hui-Li Wang

**Affiliations:** 1 Engineering Research Center of Bio-Process, Ministry of Education, Hefei University of Technology, 193 Tunxi Road, Hefei 230009, China; 2 School of Food Science and Engineering, Hefei University of Technology, Hefei 230009, China; 3 Xinjiang Shihezi Garden Dairy Co., Ltd., Shihezi 832199, China

**Keywords:** lactoferrin, EPA, emulsion, dynamics, single molecule

## Abstract

Although eicosapentaenoic acid (EPA) as a functional fatty acid has shown significant benefits for human health, its susceptibility to oxidation significantly limits its application. In this study, we developed a nanoemulsion of the lactoferrin (LTF)–EPA complex and conducted a thorough investigation of its macro- and molecular properties. By characterizing the emulsion with different LTF concentrations, we found that 1.0% LTF formed the most stable complex with EPA, which benefited the formation and stability of the emulsion against storage and freezing/thawing treatment. As the foundation block of the emulsion structure, the binding mechanism and the entire dynamic reaction process of the complex have been fully revealed through various molecular simulations and theoretical calculations. This study establishes a comprehensive picture of the LTF–EPA complex across multiple length scales, providing new insights for further applications and productions of its emulsion.

## 1. Introduction

Eicosapentaenoic acid (EPA) is a polyunsaturated fatty acid present in fish oil that can decrease the incidence and mortality associated with cardiovascular diseases, enhance anti-inflammatory and antioxidant effects, and decrease blood lipid levels [1]. However, due to its multiple double bonds, EPA is highly susceptible to oxidation, leading to the formation of harmful trans fatty acids and unpleasant odors, which significantly limits its application in food products [2]. Therefore, it is urgent to develop an effective system to protect EPA from oxidation for various beverages, milk, infant formulas, dairy-based goods, mayonnaises, dressings, as well as pharmaceuticals, cosmetics, and personal care products. Emulsions, produced with high-energy methods involving specialized mechanical devices capable of generating strong mechanical forces, offer a promising solution for realizing this purpose [3,4]. Emulsion systems based on natural biopolymers such as proteins and polysaccharides have attracted great attention due to their environmentally friendly, green, and sustainable characteristics, showing significant potential in the food, cosmetics, and pharmaceutical industries [5]. Such emulsions consist of oil droplets dispersed within a continuous aqueous phase and stabilized by surface-active molecules, which were named oil-in-water (O/W) emulsions. Several studies have demonstrated that the adsorption properties and distribution of the oil–water interface are key factors in regulating the oxidation process, specifically in protein-stabilized O/W emulsions. Natural proteins, extracted from animal or plant sources, can form an interfacial film that serves as a physical barrier and interferes with the interactions among lipids and pro-oxidants [6]. Kiokias et al. indicated that a thicker protein interfacial film could decrease the droplet size and increase the oxidative stability of emulsions [7]. Gumus et al. reported that the mechanism by which proteins affect lipid oxidation mainly lies in two aspects: on the one hand, proteins could sterically hinder the ability of pro-oxidants to interact with lipid droplet surfaces; on the other hand, proteins can protect the lipid core through self-sacrifice; i.e., the proteins preferentially react with pro-oxidants and then oxidize [8]. However, emulsions prepared based on plant-based proteins are sensitive to changes in pH and shear [9]. The size of oil droplets decreases during the formation of O/W-type emulsions with an increase in surface area, which accelerates the lipid oxidation in O/W emulsions, leading to the deterioration of the emulsions and seriously affecting their shelf life [10].

Lactoferrin (LTF) is an iron-binding globular protein derived from mammalian milk, consisting of approximately 700 amino acid residues and having a molecular weight of about 80 kDa [11]. Structurally, LTF comprises two spherical domains, the C-lobe and N-lobe, resulting in two thermal denaturation peak temperatures at 61 °C and 90 °C. The surface of LTF is often modified by glycosylation, which contributes to its structural integrity during digestion [12]. Additionally, LTF has a relatively high isoelectric point (pH 8 to 9), indicating that it carries a positive charge across a wide range of pH conditions. This property enables LTF to form complexes with anionic polysaccharides and fatty acids through hydrophobic and hydrogen bonding interactions, thereby exhibiting excellent physicochemical properties [13]. Recent advancements include the combination of LTF with epigallocatechin gallate and hyaluronic acid through a self-assembling process, resulting in the development of 145 nm nanoparticles with higher encapsulation efficiency for curcumin. This significantly enhanced the stability of curcumin against light, heat, and storage conditions [14]. Furthermore, Davi et al. explored the synthesis of LTF–triglyceride lipids with a polycrystalline surface, facilitating the incorporation of hydrophobic bioactive substances and preventing the degradation of β-carotene during storage [15]. Over the past few decades, great efforts have been undertaken to study the oxidization inhibition of lipid emulsions. However, the microscopic mechanism involved in the binding mechanism of LTF–lipid emulsions remains unclear.

Molecular dynamics (MD) simulation provides a powerful method for the microscopic mechanisms of emulsion formation and stabilization. With dramatic advances in computing power, MD simulations can be used to study complex dynamic processes in biological systems, including configuration changes, molecular folding, and molecular recognition. Moreover, MD simulations are routinely used in studying biomolecules and their complexes, specifically their structure, dynamics, and thermodynamics.

The research aim of this work is to prepare LTF–EPA emulsions for the food and pharmaceutical industries and to unravel their binding/unbinding mechanisms via a combination of various molecular simulations and theories, including unbiased molecular dynamics (MD) simulations, dissipation-corrected targeted molecular dynamics (dcTMD) simulations, and Langevin simulations [16,17]. These advanced techniques enable not only evaluating the macroscopic properties but also exploring the single-molecule origin, which can hardly be achieved due to the difficulty in terms of characterization resolution and strategy. The MD simulations reveal the binding mode and main interactions in maintaining the complex structure, indicating a strong binding energy between the ligand and protein. Treating the binding and unbinding processes as biological reactions, the dcTMD and Langevin simulations further provide the reaction pathway and reaction rate of both the binding/unbinding processes, thereby constructing the entire reaction landscape. Altogether, these results not only provide a molecular picture regarding complex stability but also reconstruct the reaction mechanism. These cross-scale findings provide new insights for further application and large-scale production of LTF–EPA formulations and help in designing new types of formulations from the molecular level.

## 2. Materials and Methods

### 2.1. Materials

LTF was obtained from Shanghai Yuanye Bio-Technology Co., Ltd. (Shanghai, China, CAS number, 146897-68-9). The protein purity was 95% (*w*/*w*). EPA (≥95.0%) was purchased from Macklin (Shanghai, China, CAS number, 10417-94-4). All chemicals and reagents were analytically pure.

### 2.2. Emulsion Preparation

First, dissolve lactoferrin in DI water and sonicate at 25 °C for 10–15 min. Refrigerate the solution at 4–6 °C for 10–12 h to obtain the aqueous phase. The volume ratios of lactoferrin to water used were (0.2%, 0.6%, 1.0%, 2.0%, and 3.0%) (*w*/*v*). Then, use EPA as the oil phase and shear with a high shear homogenizer (IKA, Staufen, Germany) at 25 °C (10,000–12,000 rpm) for 3 min to form a stable oil-in-water emulsion system. The nanoemulsion with 5% (*w*/*w*) EPA is present in the final emulsions.

### 2.3. Emulsion Particle Characterization

The particle size distributions and zeta potential of LTF–EPA nanoemulsions were both determined with a Zetasizer Nano-ZSE (Malvern, UK) at 25 °C according to [18].

### 2.4. Confocal Laser Scanning Microscopy (CLSM)

The microstructures of LTF–EPA emulsions were captured by confocal laser scanning microscope (CLSM, Olympus FV 1200-BX61, Tokyo, Japan). Further, 1 mg/mL Nile Red solution was used to dye the oil phase, while 1 mg/mL Nile Blue solution was used for proteins, and 5 μL of prepared samples was loaded on the glass slide for CLSM images under individual excitation wavelengths of 488 nm and 633 nm.

### 2.5. Emulsifying Characteristics

Fifty μL of each emulsion at different concentration levels was diluted 100-fold with 0.1% sodium dodecyl sulfate. The initial absorbance (*A*_0_) was recorded at 500 nm using a TU-1800 UV–Vis spectrophotometer (Beijing General Analysis, Beijing, China). The emulsification activity index (*EAI*, m^2^/g) was calculated as follows:

$$EAI(m^{2}/g)=(2\times 2.303\times A_{0}\times N)/[\rho \times \varphi \times (1-\theta )\times 10,000]$$

where *N* is the dilution factor (100) and *θ* is the oil phase ratio (0.05).

### 2.6. Stability Characteristics

Ten mL of LTF–EPA emulsions were transferred into glass tubes and subjected to 7 cycles of freezing/thawing treatments for cold stability test according to [19]. Then, the thawed emulsions were detected by a Zetasizer Nano-ZSE (see Section 2.3) and then calculate the particle size and zeta potential.

The measurement of Thiobarbituric Acid Reactive Substances (TBARSs) quantifies secondary oxidation products. In short, 1.0 mL of emulsion is mixed with 4.0 mL of Thiobarbituric Acid Reactive Substances (TBARSs) reagent in a 10 mL centrifuge tube and boiled for 30 min. After cooling to 25 °C, 1 mL trichloromethane is added to 1 mL of the sample, followed by vortexing for 2–4 min. Finally, absorbance is measured at 532 nm. The final TBARS values were calculated according to a 1,1,3,3-tetraethoxypropane standard curve.

The main oxidation product is lipid hydroperoxide. In brief, 1.0 mL of the sample solution is transferred into a dry 10 mL colorimetric tube. One drop (approximately 0.05 mL) of prepared ferric chloride solution is added, followed by dilution with chloroform–methanol solvent (7:3) up to the mark. The mixture is then thoroughly mixed. Subsequently, one drop (approximately 0.05 mL) of potassium thiocyanate solution (300 g/L) is added and mixed well. After waiting for 5 min at 25 °C, transfer the solution to a 1 cm cuvette and measure the absorbance at 500 nm wavelength using chloroform–methanol (7:3) as the reference. The results are reported as peroxide values (POVs).

### 2.7. Unbiased Molecular Dynamics Simulations

The bovine LTF structure was obtained from RCSB protein data bank (2B65) with 1.5 Å resolution [20]. The best docking conformation of LTF–EPA complex was obtained via Autodock Vina 1.2.5 [21] and then assigned with Charmm36 force field [22] for the following simulations on Gromacs 2020.6 [23]. In brief, the docked structure was placed in a periodic box (7 × 7 × 7 nm^3^) filled with 8933 TIP3P water molecules, treated by energy minimization, NVT and NPT equilibrium (the system was gradually heated from 0 K to 300 K during the equilibration phase using a V-rescale thermostat), followed by 100 ns unbiased simulations with a timestep of 2 fs to capture the fully stable structure of the complex with 100 configurations.

During simulations, the hydrogen bonds (3.5 Å and 30°) [24] and hydrophobic interactions (4.5 Å) between LTF and EPA were calculated via a homemade Python script based on PLIP tool along the MD trajectory [25]. The binding energy of LTF–EPA was calculated by MMPBSA method [26] by taking the last 10 ns frames (where the conformation became stable) as average.

### 2.8. Dissipation-Corrected Targeted Molecular Dynamics (dcTMD) Simulations

The dcTMD simulations developed by Wolf and Stock [17] were carried out based on the obtained final conformation in unbiased simulations. There, EPA was removed from the LTF (i.e., unbinding) against its center of mass by utilizing a constraint with a pulling velocity of 0.001 nm/ps and a stiffness of 1000 kJ/mol/nm^2^. At least 100 individual pulling events were collected for obtaining the statistically significant results (pulling work). Then, the friction coefficient, the friction-consumed energy, and the free energy were extracted from a nonequilibrium Langevin equation.

### 2.9. T-Boosted Langevin Simulations

The temperature-boosted Langevin simulations were then used to estimate the binding and unbinding rates based on a homemade C++ Script with temperature ranging from 400 to 900 K at intervals of 100 K. Here, EPA was regarded as a single abstractive particle that diffuses inside/outside the LTF under thermal fluctuations. For each temperature, 1000 ns simulation was carried with a timestep of 5 fs. The distance between LTF and EPA below 0.50 nm was regarded as binding state and above 1.0 nm was regarded as unbinding state. The rate at room temperature (~300 K) was estimated based on the above results according to the Kramers-type equation. Detailed theoretical background can be found in [16,27].

### 2.10. Statistical Analysis

All experiments were conducted in triplicate. Significant differences among samples were analyzed using one-way ANOVA with Duncan’s test (*p* < 0.05) via SPSS16 (SPSS Inc., Chicago, IL, USA). Spectral drawings were performed using Origin 8.5 (Origin Lab Corporation, Northampton, MA, USA). The replication of simulations was listed as follows: For the unbiased MD simulations, at least 3 simulations (100 ns each) were carried out in parallel to validate the robustness of the binding interface. In the MMPBSA energy calculations, 100 structures in different time nodes were collected to evaluate the mean binding energy with standard deviation. Then, in the dcTMD simulations, at least 100 efficient independent pulling experiments were performed to collect largely sampled results, where the average work conducted during the pulling process was estimated. In the T-boosted Langevin calculations, at least 3000 binding/unbinding events at each temperature were monitored for reaching a reliable analysis of the binding/unbinding rate.

## 3. Results and Discussion

### 3.1. Morphology of Different LTF–EPA Emulsions

**Figure 1:**
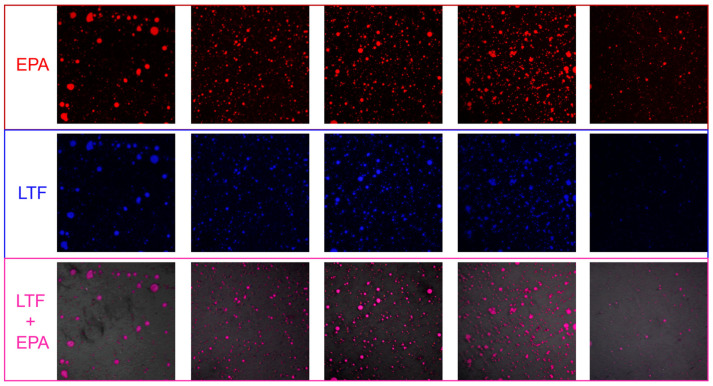
CLSM images of different ratios of LTF–EPA emulsions at 20 × magnification.

A CLSM is a powerful tool to visualize the interface structure of emulsions and the distribution of the droplet size [28]. The effects of different ratios on the microstructures of LTF–EPA emulsions (Figure 1) clearly show that the blue oil phase is distributed within the red LTF matrix. The droplet size distribution is uniform, transitioning from larger to smaller droplets in the 0.2–1% LTF–EPA groups. This suggests that a stable interface between LTF and EPA is formed by LTF adsorption onto the surface of EPA globules during the homogenization process, thereby preventing the aggregation of EPA globules and achieving a homogenized emulsion. However, when the concentration of LTF exceeds 2%, the LTF particles at the oil–water interface become more hydrophobic and tend to disperse in the aqueous phase, failing to properly incorporate around the EPA.

### 3.2. Characteristics of Different LTF–EPA Emulsions

**Figure 2:**
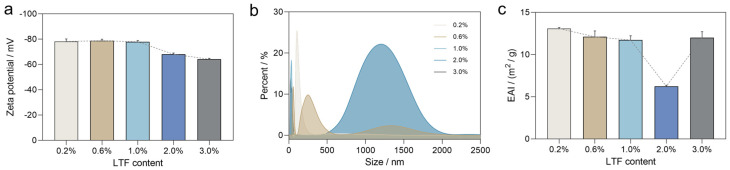
(**a**) The zeta potentials, (**b**) particle size distributions, and (**c**) emulsification activity index values of different ratios of LTF–EPA emulsions. The error bar indicates the standard deviation for at least 3 parallel measurements.

Zeta potential refers to the charge of the shear plane on particles, serving as an indicator of the stability of colloidal dispersion systems. An absolute value <30 mV typically means that the charged particles are unstable and prone to aggregation. As shown in Figure 2a, the zeta potential of all the sample emulsions is negative (absolute value >30 mV), indicating that the emulsion is relatively stable and resistant to aggregation. At low LTF–EPA concentrations (<1%), there is no significant difference in the absolute value of the zeta potential, suggesting strong interactions or attractions between the particles in the emulsion. However, this value gradually decreases as the LTF concentration approaches 2%. This fluctuating change is also evident in Figure 2b, which shows a significant peak at a particle size of around 1200 nm. With increasing LTF addition, the distribution curves exhibit a regular right shift. Consequently, the particle size distribution curve of the LTF–EPA emulsion displays single peaks at low concentrations and double peaks at high concentrations. Specifically, the 1% LTF–EPA emulsion has a higher absolute value of zeta potential and smaller homogeneous oil droplets due to the strengthened electrostatic repulsion in the system. This enhances EPA encapsulation by LTF or its physical embedding within the protein matrix, resulting in a more uniform and concentrated emulsion particle. Conversely, higher concentrations lead to more LTF aggregation into larger particles within the emulsion. Previous studies have indicated that smaller particle sizes cause dense layer formation at the interface of droplets and prevent their coalescence [29]. Here, we conducted an Emulsifying Activity Index (EAI) experiment on different ratios of LTF–EPA emulsions (Figure 2c). Among all the samples, the EAI value remained almost unchanged (11–13) within the 0.2–1.0% range but significantly decreased to 6 at 2.0%, likely due to protein aggregation from the high concentration. At a high LTF content, the absolute value of the zeta potential was decreased, indicating a weakening of the electrostatic repulsion [30]. It was crucial for the stability of the particle suspension and inhibiting the formation of stable emulsions, resulting in lower EAI values compared to other groups. This observation is consistent with the changes observed via the CLSM, supporting the conclusion that excess LTF disrupts stable emulsion formation.

### 3.3. Storage Stability of Different LTF–EPA Emulsions

**Figure 3:**
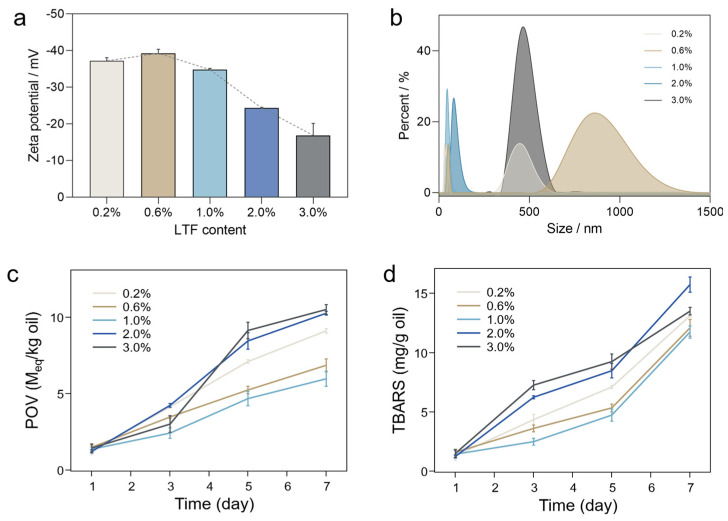
(**a**) The zeta potential values, (**b**) particle size distributions of different LTF–EPA emulsions after 7 cycles of freezing/thawing treatment, and oxidation stability including (**c**) POVs and (**d**) TBARSs during 7 days of storage. The error bar indicates the standard deviation for at least 3 parallel measurements.

As a common preservation method for food products, freezing is widely utilized in ready-to-eat foods for extended shelf life by inhibiting the growth of microorganisms. It typically requires thawing by traditional heat or microwave before consumption. However, the freeze–thaw process often results in reduced quality of food products, including appearance, texture, mouthfeel, flavor profile, etc. Thus, we further evaluated the stability of the emulsion after the freezing/thawing treatment (seven cycles), where the zeta potential and particle size distribution were as indicated (Figure 3a,b). After the treatment, the zeta potential of low concentrations (<1.0%) slightly decreased, while the particle size showed a right shift. A dual peak curve regarding the particle size distribution was observed at 0.2% and 0.1% LTF contents, indicating poor stability in low-temperature environments. However, the average diameter of the particles in the 1% LTF–EPA dispersions remained consistently around 45 nm. This stability diminishes with higher concentrations, suggesting that 1% LTF–EPA emulsions are more stable. Previous studies have shown that the partial coalescence process of EPA causes oil globules to aggregate during freezing treatment due to its crystalline phase [31]. In the case of LTF–EPA emulsions at 0.2% and 0.6%, the insufficient adsorption of LTF on the EPA surface led to partial coalescence at the oil interface, resulting in larger individual oil globule sizes. These results are consistent with the oxidative stability analysis (Figure 3c,d). Most of the lipids consumed by the population are in the form of oil-in-water (O/W) emulsions and play a crucial role by being the location where lipids, oxygen, and pro-oxidants enter into contact. The large interfacial area typically found in emulsions promotes the exposure of lipids to pro-oxidant agents and thus accelerates lipid oxidation compared to bulk oils [32]. The addition of LTF eliminated the formation of secondary products (TBARSs) and lipid oxidation of primary (POVs) under 1% concentrations, indicating better oxidation stability during storage. At 1%, the POV and TBARS values after 7 days of storage were 6.0 M_eq_/kg and 11.7 mg/g, respectively. LTF-based interfacial layers promoted the steric hindrance between the aqueous phase and the lipid phase, further inhibiting the lipid oxidation to a certain extent and improving the oxidation stability [33,34]. The frozen storage analysis can increase the tenability of LTF–EPA emulsions and create possible applications in frozen food products.

Based on the above analyses, the 1% LTF–EPA emulsion showed better characteristics than the other groups. For example, although the 3% LTF–EPA had the highest concentration of LTF, the POV was much lower (10.5 M_eq_/kg) than in the 1% group (6.0 M_eq_/kg), and the TBARS value was increased from 11.7 to 13.5 mg/g. It is worth noting that the stability of the complex may be challenged by environmental variations, such as the changes in pH and temperature that often occur during storage. Therefore, we have further explored the stability via the zeta potential and particle size distribution (Supplementary Figure S1). It was found that the particle size of LTF–EPA significantly decreased with a decrease in pH, which indicated a potential use in pH-sensitive switchable biocomponent delivery. Meanwhile, the LTF–EPA emulsion remained almost unchanged at the range of 50–70 °C but became aggregated at 80 °C. Combined with the zeta-potential change analysis, the high temperature changed the protein structure and weakened the electrostatic repulsion and hydrogen bonding between the particles, which caused the aggregation of the emulsion droplets [35]. In addition, the FTIR spectra showed that the positions of the peak apexes of the LTF–EPA emulsions did not change significantly, and no new absorption peaks appeared or disappeared (Supplementary Figure S2). This suggests that blocking the generation of hydrophobic interactions may not completely change the structure of the protein or cause it to generate new functional groups within the emulsion. The broad peaks at 3200 cm^−1^ are stretching vibrations of O-H and N-H, indicating the existence of inter- and intramolecular hydrogen bonds inside the emulsion system.

To estimate the release efficiency of EPA in vivo, we conducted in vitro digestion experiments under simulated digestion conditions over 120 min (Supplementary Figure S3). The results showed that, under the 1% LTF condition, EPA had the lowest release efficiency, indicating that the EPA–LTF complex was more effectively maintained in this case, which provided better protection for EPA against digestion.

### 3.4. The Molecular Interface of the Binding Complexes

**Figure 4:**
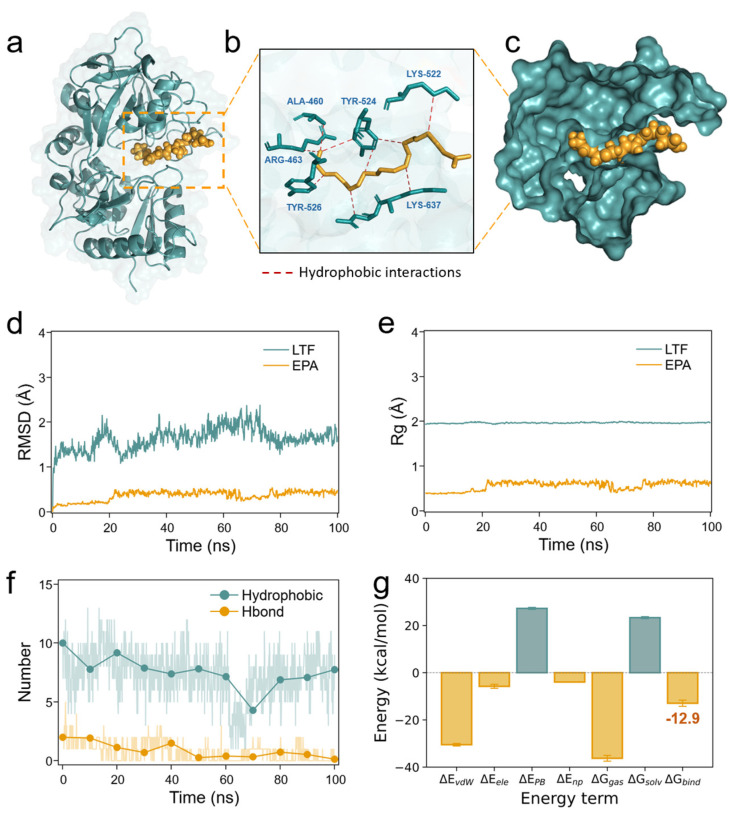
Unbiased MD simulations of the LTF–EPA complex (**a**) for 100 ns show that EPA could bind to the LTF protein via (**b**) hydrophobic interactions among the carbon backbone and several hydrophobic residues, forming a hydrophobic binding pocket with a sectional view shown in (**c**). (**d**,**e**) The RMSD and Rg of both the protein and ligand during simulations. (**f**) Analysis of the number of hydrophobic interactions and hydrogen bonds between LTF and EPA with a (**g**) binding energy of −12.9 kcal/mol calculated via MMPBSA method.

In the section above, we demonstrated that LTF can bind to EPA and form a stable complex in certain conditions. In the following, we utilized single-molecule theoretical and simulation methods to deeply investigate the molecular details of the LTF–EPA complex. First, 100 ns unbiased MD simulations were carried out to reveal the molecular interface of the binding complex (Figure 4a–c). It can be seen that EPA, as a long linear molecule, fits well into the active pocket of LTF due to their matching dimensions, enabling it to insert and bind effectively. Apart from the terminal carboxyl group, the entire backbone of EPA is hydrophobic; thus, it primarily interacts with the hydrophobic pocket through hydrophobic interactions and extends its hydrophilic carboxyl tail into the water. Such protection of EPA within LTF could significantly prevent the oxidation of EPA. To further examine the binding affinity of the two, we then characterized the changes in molecular conformation and size using root mean square deviation (RMSD) and radius of gyration (Rg). Throughout the simulation process (Figure 4d,e), the structures of both LTF and EPA did not undergo significant changes, indicating that their binding is quite stable. This stability is mainly attributed to multiple hydrophobic interaction sites (7.3 ± 2.1 sites), while the contribution of hydrogen bonds is negligible (<0.8 sites). Such interactions endow the complex with a binding energy of −12.9 kcal/mol (estimated using the MMPBSA method, which summarizes the energy contributions from molecular mechanics and solvation effects, including both polar and non-polar interactions). This result is comparable to the binding energy of classical protein–ligand systems [16], indicating a robust pairing formation in our case. The results above can be further summarized as follows: EPA can bind to the LTF pocket because both are highly non-polar and have compatible sizes for mutual nesting. After binding, EPA can stably remain in the pocket of LTF because their specific interactions hold them together.

### 3.5. The Binding/Unbinding Dynamics of the Complex via Dissipation-Corrected Targeted MD and T-Boosted Langevin Simulations

**Figure 5:**
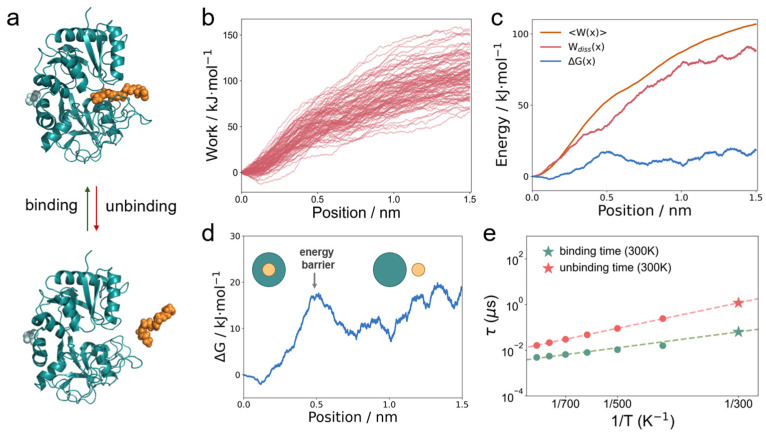
(**a**) Estimation of the binding/unbinding dynamics of LTF–EPA via dcTMD simulations. (**b**) The consumed work trajectories from 100 constraint pulling events. (**c**) *<W(x)*>, W*_diss_*(x), and Δ*G(x)* along the pulling coordinate. (**d**) The free energy profile indicates the energy barrier at ~0.5 nm as a boundary of binding/unbinding. (**e**) The binding and unbinding times as a function of temperature.

In the following, we further explore the dynamics of the complex via a theoretical method approach by solving the binding/unbinding rate numerically based on the Langevin equation. Due to the tight binding of EPA in the active pocket of LTF, the events of unbinding and rebinding are very rare [36], posing challenges for further study of its reaction kinetics in unbiased simulations. Here, we investigate the dynamic binding and unbinding processes of EPA and LTF (Figure 5a) using the dissipation-corrected TMD method [17]. Here, a constraint force *f_c_* with a pulling velocity *v_c_* was applied to the EPA against the LTF, which gradually pulled it out of the pocket until complete dissociation was achieved, thereby realizing force-accelerated unbinding. We collected at least 100 independent pulling experiments and calculated the average work conducted during the pulling process (i.e., the energy injected by the external force, Figure 5b). During the pulling process, *f_c_* applied a constraint to the EPA molecule and caused it to move following *x = x_0_ + v_c_t*. This nonequilibrium kinetic process can thus be described by the Langevin equation, where the EPA suffers friction and collision along the pulling coordinate:

$$m\ddot{x}(t)=-\frac{dG}{dx}-\gamma \left(x\right)\dot{x}+\sqrt{2k_{B}T\gamma \left(x\right)}\xi \left(t\right)+f_{c}(t)$$

where *G* is the free energy, γ is the friction coefficient, and 〈*ξ*〉 = 0 is the delta-correlated collision noise. In the case of pulling at a constant *v_c_*, the acceleration of the EPA molecule vanishes. Thus, the above equation can be written for the averaged results over multiple pulling experiments as

$$\Delta G\left(x\right)=<W\left(x\right)>-v_{c}\int_{x_{0}}^{x}\gamma \left(x^{\prime }\right)dx\prime$$

We included the average pulling work *<W(x)>*, the dissipation work *W_diss_(x),* and the free energy profile Δ*G(x)*, whose results were calculated and are shown in Figure 5c. By closely examining Δ*G(x)* along the pulling coordinate (Figure 5d) [37], we observed a significant energy barrier when the LTF–EPA distance was at 0.5 nm, which impedes the motion of EPA across this threshold. This barrier can be considered as the boundary condition for LTF–EPA binding/unbinding: they remain bound when the distance is less than 0.5 nm and dissociate rapidly once the distance exceeds 0.5 nm.

With all the molecular dynamics information obtained, it becomes possible to calculate the binding/unbinding rates according to the single-molecule theories. Using the aforementioned equations, we were able to perform μs-scale coarse-graining computation steps at a given temperature and analyze the LTF–EPA distance changes to derive statistically significant rate results. Due to the insufficiently vigorous motion of EPA at room temperature (300 K), observing a sufficient number of binding/unbinding events remains challenging. Thus, we utilize the T-boosted Langevin equation [16] to compute the rate *k*_1_ at higher temperatures (400–900 K) and then extrapolate the rate *k*_2_ at room temperature using the Kramers-type equation:

$$k_{2}=k_{1}e^{-\Delta G(\frac{1}{k_{B}T_{1}}-\frac{1}{k_{B}T_{2}})}$$

From Figure 5e, we find that the dissociation time *τ* (i.e., the inverse of the rate) at room temperature can reach up to 1 μs, whereas the binding time is only about 0.01 μs. Note that this duration significantly exceeds the time window of unbiased simulations in the nanosecond range, thus making observation difficult. This indicates that the LTF–EPA binding is quite stable, with the binding rate being nearly 100 times the dissociation rate. Altogether, we could reconstruct the whole dynamic process of LTF–EPA, including the binding/unbinding rate, energy profile, and energy dissipation. These results thus provide a thorough theoretical understanding of the dynamics of the LTF–EPA complex. It is worth noting that the calculation of the binding and unbinding rates is very difficult in common MD methods since it is not possible to monitor sufficient binding events at the nanosecond scale. An alternative method is to calculate the rate via physical laws such as the Langevin equation, which requires knowledge of the energy and friction profiles to fully describe the movement of the ligand. By using the dcTMD approach, these parameters can be well extracted from the MD simulations and applied to the Langevin equation. Nonetheless, binding events remain very rare because, at 300 K, the movement of molecules is not drastic. Therefore, the T-boosted Langevin equation is used to capture sufficient events at higher temperatures where the movement of molecules is drastic. Then, the rate at 300 K can be extracted from the higher-temperature results by the Kramers-type equation (altogether, a combination of dcTMD and T-boosted Langevin).

It is important to bridge the gap between these molecular results and macroscopic data as they are governed by physical laws at very different length scales. The connection between the two can be classified into two points: (i) the binding between EPA and LTF is driven by hydrophobic interactions, where EPA happens to be positioned in a linear structure that fits into the cavity of LTF. This is reflected at the macroscopic level as LTF is adsorbed at the droplet interface with EPA, showing a high zeta potential and a smaller particle size distribution. (ii) EPA exhibits a binding time nearly 100 times shorter than the dissociation time, indicating that EPA strongly favors forming a complex with LTF rather than dissociating, meaning the reaction strongly proceeds in the direction of complex formation. This is reflected at the macroscopic level as EPA prefers to interact with LTF rather than dissociate, thereby forming a viscous emulsion that is resistant to storage and freezing/thawing treatments. These findings can further guide the design of new composite emulsions based on LTF: hydrophobic ligands and ligands with linear structures can effectively bind with LTF to form complexes, and improving the hydrophobic properties of the ligands can enhance binding and inhibit dissociation.

## 4. Conclusions

By investigating emulsions with different LTF concentrations, we found that 1.0% LTF forms the most stable complex with EPA, which decreases the average particle size and improves surface charge, dispersibility, and emulsion activity. This concentration results in the best formation and stability of the emulsion against storage and freezing/thawing treatments. Molecular simulations and theoretical analyses further reveal that the binding rate is significantly higher than the unbinding rate during the dynamic process, providing a robust structure with high stability. These results offer a comprehensive understanding of the binding process between LTF and EPA, including the binding affinity, molecular properties, and the characteristics of the resulting complexes. However, such O/W emulsions stabilized by protein may be more sensitive to digestion than protein–polysaccharide emulsion. For instance, they may gradually dissociate in a reversible manner under the stimulation of gastrointestinal digestive juice, which cannot maintain the structural stability of emulsion in the digestive tract and achieve the purpose of inhibiting lipid digestion. The protein–polysaccharide composite interfacial structure can be rationally designed based on the intermolecular interaction between proteins and polysaccharides in the manner of hydrophilic and hydrophobic complementarity so as to improve the resistance of the protein–polysaccharide composite interfaces to the external environment. Thus, in future research, we will thoroughly investigate the in vitro digestion behaviors between the LTF–EPA complex and LTF–polysaccharide–EPA complex and determine their differences.

## Data Availability

The original contributions presented in this study are included in the article/Supplementary Material. Further inquiries can be directed to the corresponding author.

## Supplementary Materials

The following supporting information can be downloaded at https://www.mdpi.com/article/10.3390/foods14010082/s1. Figure S1: zeta potential and particle size distribution of 1% LTF-EPA emulsions under different pH and temperature. Figure S2: FTIR results of EPA, LTF and 1% LTF-EPA complex. Figure S3: EPA release of different LTF-EPA emulsions in simulated digestion conditions.
