# Supplementary material for: Dynamics and Stability Mechanism of Lactoferrin–EPA During Emulsification Process: Insights from Macroscopic and Molecular Perspectives
Source: Foods. 2025 Jan 1;14(1):82. doi: 10.3390/foods14010082 (PMC11719685; doi:10.3390/foods14010082)
Supplement: Supplementary file 1 [file foods-14-00082-s001.zip › foods-3406229-supplementary.pdf]

Dynamics and Stability Mechanism of Lactoferrin–EPA During Emulsification Process: Insights from Macroscopic and Molecular Perspectives

Han Tao [1,2], Wei Ding [2], Mengjia Fang [2], Hao Qian [3], Wan-Hao Cai [1,2,*] and Hui-Li Wang [1,2]

1 Engineering Research Center of Bio-Process, Ministry of Education, Hefei University of Technology, 193 Tunxi Road, Hefei 230009, China
2 School of Food Science and Engineering, Hefei University of Technology, Hefei 230009, China
3 Xinjiang Shihezi Garden Dairy Co., Ltd., Shihezi 832199, China
* Correspondence: caiwanhao@hfut.edu.cn (W.-H.C.)

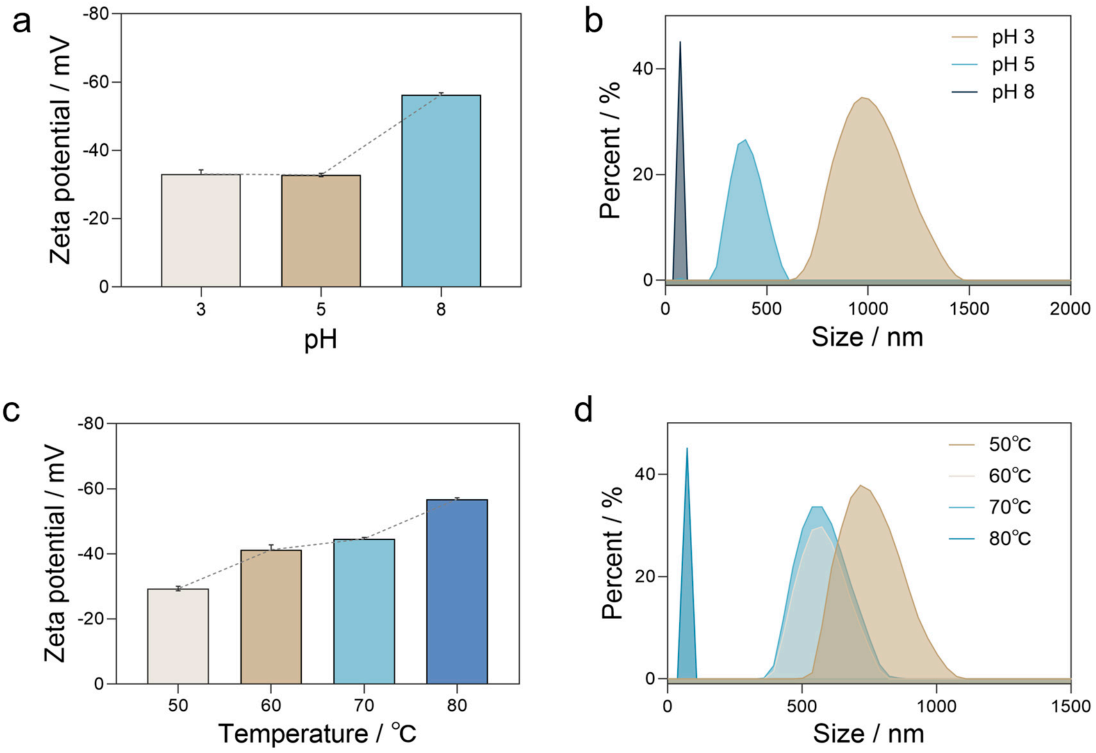

Figure S1. (**a**) The zeta potential and particle size distribution of 1% LTF–EPA emulsions under different values of (**a**,**b**) pH and (**c**,**d**) temperature.

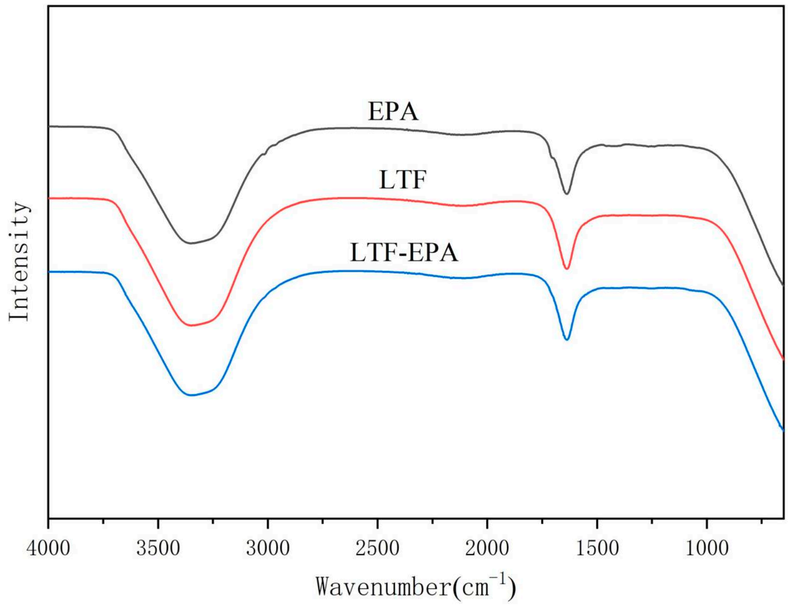

Figure S2. FTIR results of EPA, LTF, and 1% LTF–EPA complex.

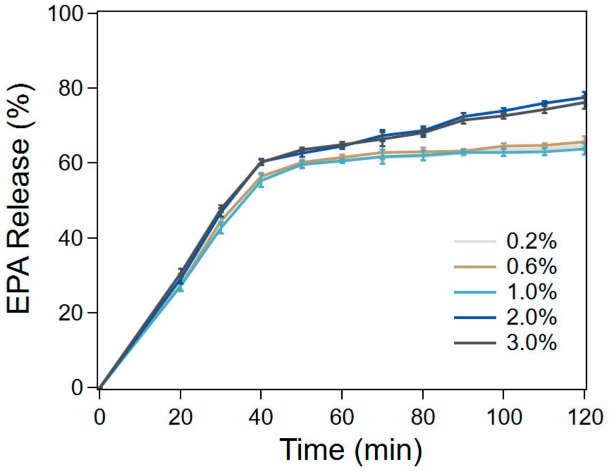

Figure S3. EPA release of different LTF–EPA emulsions in simulated digestion conditions.

The in vitro digestion experiment consists of two processes: simulating gastric digestion and simulating intestinal digestion. The simulated gastric fluid was prepared by dissolving 1 mL hydrochloric acid, 3.2 g pepsin, and 8.77 g NaCl in 1000 mL water. The simulated intestinal fluid was prepared by dissolving 1.11 g $CaCl_2$, 20 g bile salts, and 1 g pancreatin in 1000 mL of water

and then adjusting the pH to 7.0 with 0.1 mol/L NaOH. Then, 15 mL lactoferrin–EPA emulsion was added to 20 mL simulated gastric fluid, followed by adjusting the pH of the mixture to 3.0 using 1 M HCl solution. The digestion system was placed in a water bath (37°C) and stirred with a magnetic stirrer at 100 rpm for 1 h. After that, the treated sample was added to 30 mL simulated intestinal fluid. The digestion system was also placed in a water bath (37°C) and stirred with a magnetic stirrer at 100 rpm for 1 h. Note that the pH may decrease due to the hydrolysis of EPA by lipase, where the pH needs to be monitored and maintained at 7.0 by adding 1 M NaOH. The amount of NaOH used was recorded for calculating the released free fatty acids as follows:

$$FFA = \frac{V_{NaOH} \times C_{NaOH} \times M_{lipid} \times 1000}{2W_{lipid}}$$

where V_{NaOH} and C_{NaOH} are the titration volume and concentration of the standard NaOH used; M_{lipid} is the molar mass of fatty acids; W_{lipid} is the mass of lipids in the small intestine as a whole.
